# The Effect of Static Magnetic Fields of Different Strengths and Polarities on Cytokine Production by Human Lymphocytes In Vitro

**DOI:** 10.3390/bioengineering11080749

**Published:** 2024-07-24

**Authors:** Vladimir Turuntaš, Silvio de Luka, Jasna L. Ristić-Djurovic, Saša Ćirković, Drago Djordjevich, Siniša Ristić, Nenad Lalović, Veljko Marić, Bratislav Lazić, Bojan Joksimović, Ivan Stanojevic, Saša Vasilijić, Alexander M. Trbovich

**Affiliations:** 1 University Hospital Foča, 73300 Foca, Republic of Srpska, Bosnia and Herzegovinanenad.lalovic@gmail.com (N.L.); veljko_maric@yahoo.com (V.M.); 2 Faculty of Medicine Foca, University of East Sarajevo, 73300 Foca, Republic of Srpska, Bosnia and Herzegovina; risticsinisa@yahoo.com; 3 Department of Pathological Physiology, School of Medicine, University of Belgrade, 11000 Belgrade, Serbia; silvio.de-luka@mfub.bg.ac.rs (S.d.L.); dragodj@gmail.com (D.D.); alexander.m.trbovich@yahoo.com (A.M.T.); 4 Institute of Physics Belgrade, National Institute of the Republic of Serbia, 11060 Beograd, Serbia; jasna@stanfordalumni.org (J.L.R.-D.); kosjera@ipb.ac.rs (S.Ć.); 5 Faculty of Medicine, University of Pristina Temporarily Settled in Kosovska Mitrovica, 38220 Kosovska Mitrovica, Serbia; bratislav.lazic@yahoo.com; 6 Institute for Medical Research, Military Medical Academy, 11000 Belgrade, Serbia; ivanivanstanojevic@gmail.com (I.S.); vasilijics@yahoo.co.uk (S.V.); 7 Faculty of Medicine of the Military Medical Academy, University of Defense in Belgrade, 11000 Belgrade, Serbia

**Keywords:** static magnetic field, cytokines, lymphocytes

## Abstract

In contrast to electromagnetic fields, static magnetic fields (SMFs) have not been extensively studied in terms of their potential health consequences. Although upward- and downward-oriented magnetic poles may cause various biological effects, only the pole with the upward orientation has been mainly investigated. Considering that the interaction of antigen-presenting cells (APCs) and T lymphocytes is crucial to trigger an immune response, we assessed the effect of long-term exposure of human T lymphocytes and dendritic cells (DCs) to moderate strength SMFs of different orientations focusing on the cytokine profile of activated T cells. Cultures of allogenic T lymphocytes and DCs (immature and matured by TLR3 and TLR7 agonists) were continuously exposed to four SMFs. The intensity of the applied field was 1 militesla (mT) or 56 mT of the upward- and downward-oriented pole of the SMF. Cell culture supernatants were assayed for IFN-γ, IL-4, IL-17, TNF-α, TNF-β, IL-1 β, IL-6, IL-8, and IL-10 by ELISA or flow cytometry. The upward-oriented 56 mT SMF significantly increased the release of IFN-γ and TNF-β (both *p* < 0.05) in the cell culture supernatants of T cells and immature DCs. In contrast, the same cultures exposed to the upward-oriented 1 mT SMF showed significantly elevated levels of IL-17 (*p* < 0.05). The levels of IL-4, TNF-α, IL-1 β, IL-6, IL-8, and IL-10 were not affected by the upward-oriented SMF. The downward-oriented 56 mT SMF increased TNF-α release when T cells were stimulated with mature DCs. The production of other cytokines was unchanged by the downward-oriented SMF. These findings demonstrate for the first time different in vitro biological effects of upward- and downward-oriented static magnetic fields on the cytokine production of T cells activated by DCs, helping to better understand SMF effects on the immune system and suggesting that the selective SMF effect on the immune response could have potential therapeutic effects in different immune-mediated disorders.

## 1. Introduction

As science and technology have advanced, the frequency of exposure to static magnetic fields (SMFs) has significantly increased. Individuals can be exposed to an SMF during activities such as magnetic resonance imaging (MRI), nuclear magnetic resonance (NMR) spectroscopy, and travel on magnetic levitation transportation systems. While the biological effects of an SMF on human health remain poorly understood, the World Health Organization (WHO) continues to monitor for any potential impacts on individuals [1].

Researching the biological impacts of an SMF using cell cultures is a promising avenue. An SMF was classified as weak (<1 mT), moderate (1 mT to 1 T), strong (1–5 T), and ultrastrong (>5 T) based on its induction value [2]. Most studies on SMFs’ effects have focused on strong magnetic fields and acute exposures, often due to concerns about the potential negative health effects of MRI [3,4,5]. However, studies investigating the effects of a moderate SMF combined with long-term exposure are rare, especially those focusing on immune cells [6,7]. The immune system’s primary cells include leucocytes such as neutrophils, eosinophils, basophils, lymphocytes, and monocytes. Lymphocytes are further divided into three major subtypes: T cells, B cells, and natural killer (NK) cells. During the activation of the immune response, antigen-presenting cells (APCs) and T lymphocytes engage in a complex interaction [8]. Among APCs, dendritic cells (DCs) are the most potent stimulators of T cells, leading to their proliferation, differentiation, and cytokine production. Most studies examining the effects of an SMF on immune cells have utilized human peripheral blood mononuclear cells (PBMCs) and occasionally isolated lymphocytes, observing their response to mitogen-induced stimulation [9]. However, as DC and T cell interaction is crucial for the activation of naïve T cells, using DCs as activators of T cells instead of mitogens may better reflect and mimic an immune response in vivo. Furthermore, recent in vivo investigations have revealed that distinct biological effects can be caused by SMFs with different polarities (directed upward and downward) [10,11]. Therefore, we aimed to examine the effects of long-term exposure to moderate-strength SMFs (1 mT and 56 mT) of different orientations on in vitro cytokine production in cultures consisting of purified human T lymphocytes and monocyte-derived DCs. In this in vitro model that could be precisely controlled, we predict that numerous, even dichotomous, biological impacts at the level of several immunological parameters could be observed. To our knowledge, no in vitro SMF study utilizing T lymphocytes activated by DCs has been conducted so far.

## 2. Materials and Methods

### 2.1. Study Design

The study was performed as a part of the project approved by the Ethical Board of Military Medical Academy, Belgrade, Serbia (project number: MFVMA/10/13-15, permission date 12 September 2015).

**Figure 1:**
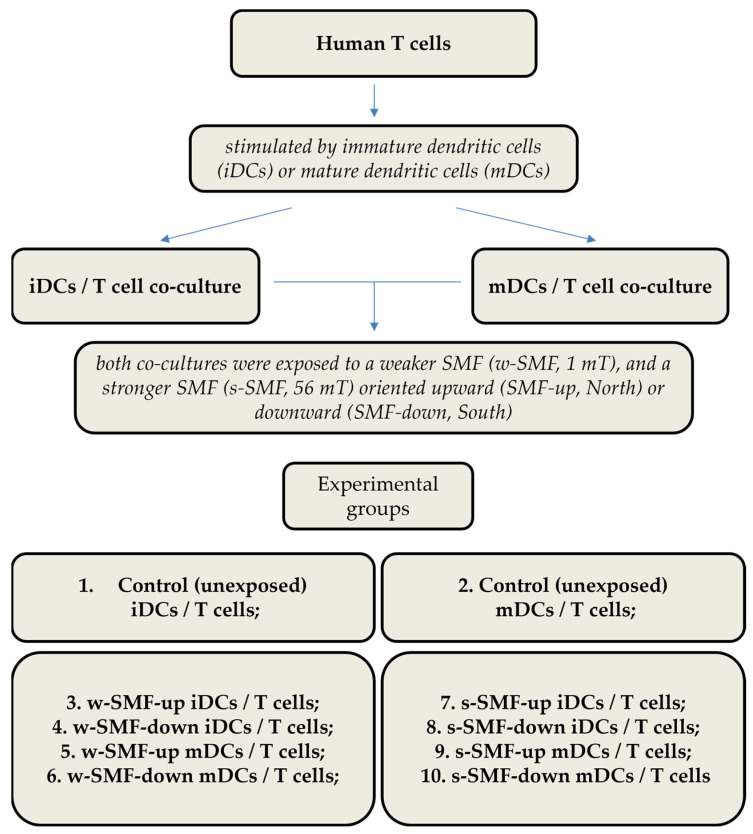
Experimental design with different groups and experimental chronology after exposure to static magnetic field.

The following experimental conditions were created: (1) control cultures composed of T cells and immature DCs (iDCs) unexposed to the magnetic field (control iDCs/T cells); (2) control cultures composed of T cells and mature DCs (mDCs) unexposed to a magnetic field (control mDCs/T cells); (3) T cells and iDCs exposed to an upward 1 mT SMF (w-SMF-up iDCs/T cells); (4) T cells and iDCs exposed to a downward 1 mT SMF (w-SMF-down iDCs/T cells); (5) T cells and mDCs exposed to an upward 1 mT SMF (w-SMF-up mDCs/T cells); (6) T cells and mDCs exposed to a downward 1 mT SMF (w-SMF-down mDCs/T cells); (7) T cells and iDCs exposed to an upward 56 mT SMF (s-SMF-up iDCs/T cells); (8) T cells and iDCs exposed to a downward 56 mT SMF (s-SMF-down iDCs/T cells); (9) T cells and mDCs exposed to an upward 56 mT SMF (s-SMF-up mDCs/T cells); and (10) T cells and mDCs exposed to a downward 56 mT SMF (s-SMF-down mDCs/T cells) (Figure 1).

SMF inductions of 1 mT and 56 mT were selected considering previous studies that have shown different effects on immune cells [12,13].

### 2.2. Cells

PBMCs from buffy coats of healthy volunteers who signed consent forms were isolated by density centrifugation on a lymphoprep gradient (Nycomed, Oslo, Norway). Plastic adherence was used to separate monocytes from PBMCs, and monocyte-derived DCs were produced from the adherent portion of PBMCs. The procedure involved resuspending PBMCs in complete Roswell Park Memorial Institute (RPMI) 1640 medium (ICN, Costa Mesa, CA, USA), supplemented with 2 mmol/L L-glutamine, 20 mg/mL gentamicin, 50 mmol/L 2-mercaptoethanol (Sigma Aldrich, Münich, Germany), and 10% heat-inactivated fetal calf serum (FCS) (ICN, Costa Mesa (Falcon, New York, NY, USA). The nonadherent cells were removed after 1.5 h at 37 °C, and the adherent cells were then cultured for the next 5 days to produce immature MoDCs in complete RPMI medium containing 100 ng/mL of recombinant human (rh) granulocyte–macrophage colony-stimulating factor (GM-CSF), specific activity 4.44 × 10^6^ UI (Leukomax; Schering-Plough, Basel, Switzerland), and 20 ng/mL of rh IL-4 (R & D Systems, Minneapolis, MN, USA). At day 5, the immature DCs were replated (5 × 10^5^ cells/mL) in the medium containing GM-CSF/IL-4 and TLR3 agonist [poly(I:C) (10 µg/mL, Sigma-Aldrich, Münich, Germany)] or TLR7 agonist (7-thia-8-oxoguanosine) (250 µM, Sigma-Aldrich, Münich, Germany) for an additional two days.

According to the manufacturer’s instructions, allogeneic CD4+ T cells were extracted from PBMCs using negative immunomagnetic sorting using CD4 isolation kits (MACS technique; Myltenyi Biotec, Bergisch Gladbach, Germany). According to flow cytometry analysis, using an EPICS XL-MCL (Coulter, Krefeld, Germany), of cells stained with an anti-CD4 antibody (Serotec, Oxford, UK), the purity of the cells was always greater than 90%. Using the MicoAlert Mycoplasma detection kit (Lonza Nottingham, London, UK), routine mycoplasma testing was performed on all cultures.

### 2.3. Allogeneic T-Cell Activation

In an allogeneic mixed leukocyte reaction (MLR), CD4+ T cells (1 × 10^5^ cells/well) and DCs (1 × 10^4^ cells/well) were grown on 96-well round-bottom cell culture plates in complete RPMI 1640 media. After five days of culture, the cells received pulses of [^3^H]thymidine (1 μCi/well; Amersham, Amersham, UK) for the last 18 h. Labeled cells were collected onto glass fiber filters, and the radionuclide’s incorporation into DNA was further quantified by β-scintillation counting (LKB-1219; Rackbeta, Turku, Finland). Results were expressed as counts per minute (c.p.m.) ± standard deviation (SD) of triplicates.

### 2.4. Cytokine Assays

Allogeneic CD4+ T cells and DCs were cultivated for five days, as described above. After five days, phorbol myristate acetate (PMA; 20 ng/mL; Sigma-Aldrich, Munich, Germany) and ionomycin (500 ng/mL; Merck, Vienna, Austria) were added to the wells. Eight hours later, the cell cultures were centrifuged, and the supernatant was harvested and kept at −20 °C until cytokine profiling. Quantification of cytokines was performed using human ELISA kits (IFN-γ, TNF-α, TNF-β, IL-1β, IL-4, IL-6, IL-8, IL-10, and IL-17; R&D Systems, Minneapolis, MN, USA) and the FlowCytomix Human Th1/Th2 11plex kit (Bender MedSystems, Vienna, Austria).

### 2.5. Cell Viability Assays

T cells (3 × 10^5^ cells/well) were grown in 96-well round-bottom cell culture plates in complete RPMI 1640 media and were exposed to 56 mT SMFs of different orientations for 6 days, the same length of time they were stimulated with DCs in the cytokine assays. After six days, the cells were harvested and stained with propidium iodide (PI) (50 µg/mL, Sigma) in hypotonic solution (0.1% sodium citrate with 0.1% Triton X-100, Sigma) and PBS (Gibco; Thermo Fisher Scientific, Inc., Waltham, MA, USA) for quantification of apoptosis and necrosis, respectively. After staining and a short incubation in the dark at room temperature, the cells were analyzed using flow cytometry. Apoptosis was quantified as the percentage of cells with hypodiploid nuclei corresponding to fragmented DNA after staining with PI in a hypotonic solution. Necrosis was quantified as the percentage of cells stained with PI in PBS, where PI-positive cells were considered as cells with damaged cell membranes.

### 2.6. Magnetic Field

Two SMF sources with different field intensities (1 mT and 56 mT) were used to study the effects of continuous exposure to an SMF. Each of the two SMF sources was set up in two ways; in the first setup, the samples were placed in the upwardly oriented SMF and in the second setup the samples were in the downwardly oriented SMF. The exposure was continuous and was initiated at the beginning of monocyte cultivation. The control was not subject to an SMF. Since the experiment was carried out in the Northern Hemisphere, the geomagnetic field is primarily downward-directed. The geomagnetic field intensity was approximately 48 microtesla (μT).

**Figure 2:**
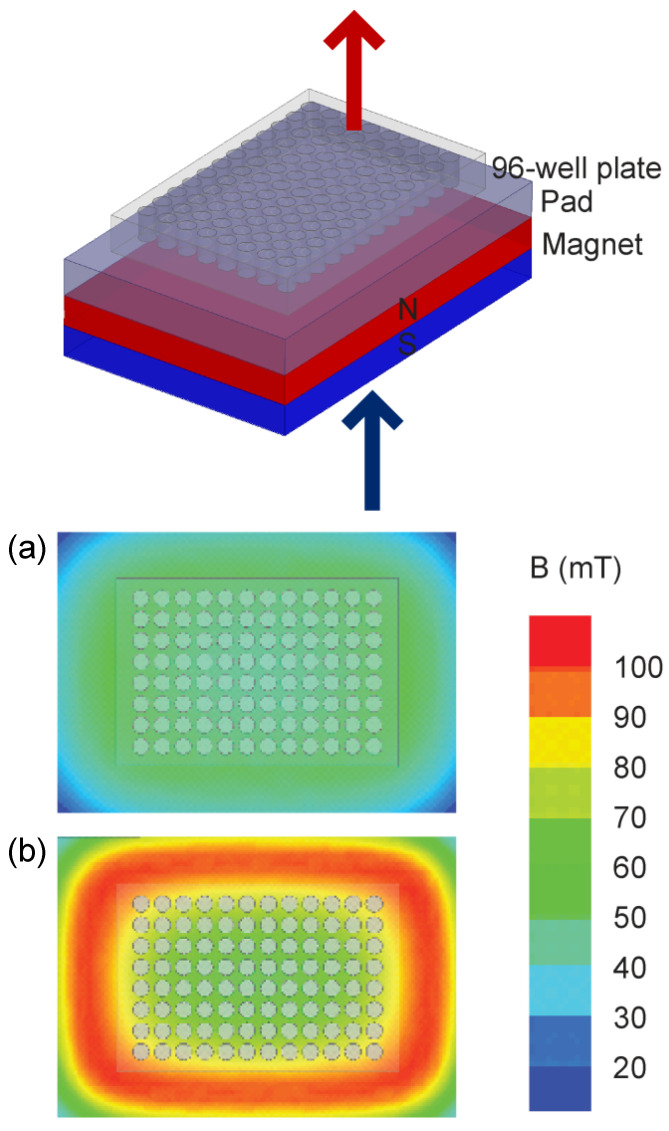
Permanent magnet. The experimental setup is shown in the upper part of the figure. The 96-well plate supported with a 1.5 cm thick plastic pad is placed on top of the 10 cm × 15 cm × 2.5 cm permanent magnet. The figure illustrates the Up group experiment, i.e., the upper pole of the magnet is the north pole and is depicted in red; consequently, the down pole is the south pole and is depicted in blue. In the Down group experiment setup, the permanent magnet was flipped so that the upper pole was the south pole and the down pole was the north pole. Note that the magnetic field is always oriented towards the south pole and away from the north pole. The 96-well plate is 8 cm × 12 cm × 1.5 cm in size. The lower part of the figure shows the magnetic field intensity. The magnetic field intensity in the top and bottom surfaces of the 96-well plate is given in panels (**a**) and (**b**), respectively. The magnetic flux intensity in the experimental volume, i.e., inside the 96-well plate, varies between its minimal and maximal values of 40.7 and 97.4 mT, whereas its average value is 56.0 mT.

**Figure 3:**
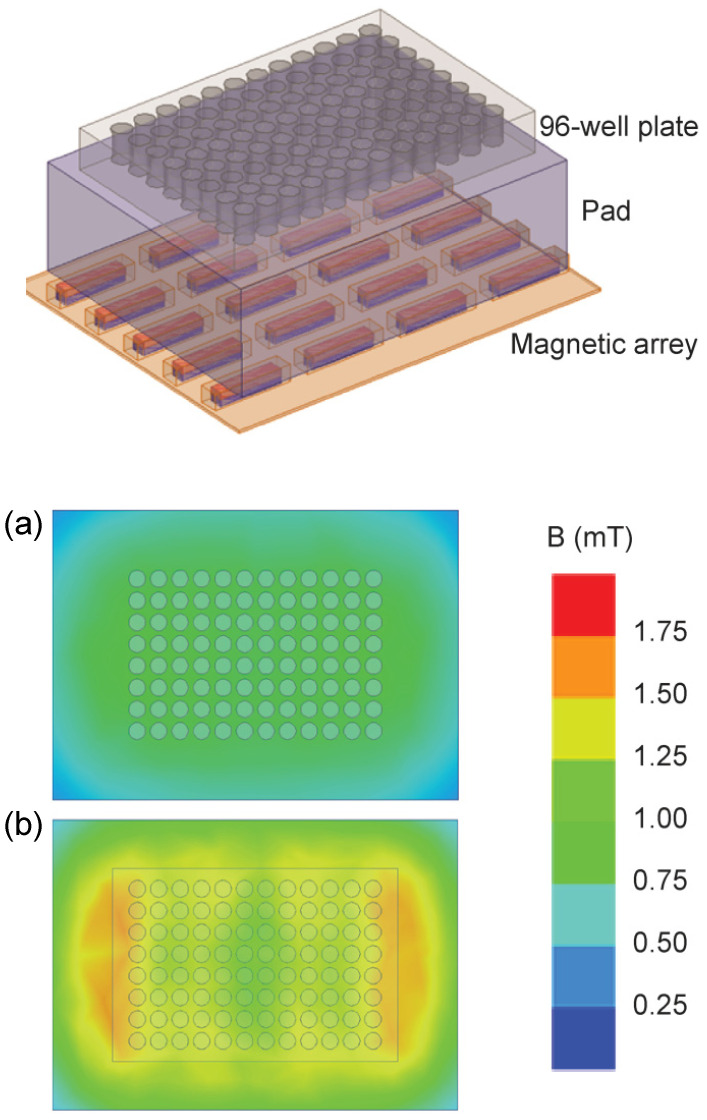
Permanent magnet array. The experimental setup is shown in the upper part of the figure. The 96-well plate with a 4 cm thick plastic pad is placed on the 4 × 5 array of permanent magnets that are each 24 mm × 5 mm × 5 mm in size. The magnets are embedded in elastic plastic. The figure illustrates the Up group experiment, i.e., the upper poles of the magnetic elements are the north poles and are depicted in red. The 96-well plate is 8 cm × 12 cm × 1.5 cm in size. The lower part of the figure shows the magnetic field intensity. The magnetic field in the top and bottom surface of the 96-well plate is given in panels (**a**) and (**b**), respectively. The minimal, maximal, and average values of magnetic flux intensity in the experimental volume are 0.6, 1.5 mT, and 1.0 mT, respectively.

Two static inhomogeneous magnetic fields were used, one originating from a single large permanent magnet (ferrite block magnet B150-100-25FA, GAUS Group, Požarevac, Serbia) and the other from an array of twenty small permanent magnets (MADU stripe, The Mihailo Pupin Institute, Belgrade, Serbia; patent number YU 48907/02, Republic of Serbia Intellectual Property Office, Belgrade, Serbia; patent number WO 99/60581, World Intellectual Property Organization, Brussels, Belgium, EU). As a result, both fields’ values fluctuate throughout the experimental volume without changing over time. The experimental setup during sample exposure to the large permanent magnet is depicted in the upper part of Figure 2, whereas the magnetic field intensities in the top and bottom planes of the setup’s experimental volume are given in the lower part of Figure 2. The illustrations corresponding to the array of permanent magnets are shown in Figure 3. The intensities of magnetic flux density given in the lower parts of Figure 2 and Figure 3 were obtained from the numerical models of the two experimental setups and were calibrated to the values that were measured in the selected representative experimental volume points. Figure 1 represents the experimental design with different groups and experimental chronologies after exposure to an SMF. Magnetic flux density measurements were conducted using a digital teslametar (DTM-151, Group3 Technology, Auckland, New Zealand) with 0.002 mT resolution. A thorough description of the permanent magnet array is given in [14].

### 2.7. Statistical Analyses

A one-way ANOVA and Kruskal–Wallis test, followed by Dunn’s multiple comparisons test, and a two-way ANOVA, followed by Bonferroni’s multiple comparisons test and a *t*-test (parametric), were performed using GraphPad Prism software (version 10.0.0 for Windows, GraphPad Software, Boston, MA, USA). All *p*-values lower than 0.05 were considered statistically significant.

## 3. Results

### 3.1. Cytokine Release

**Figure 4:**
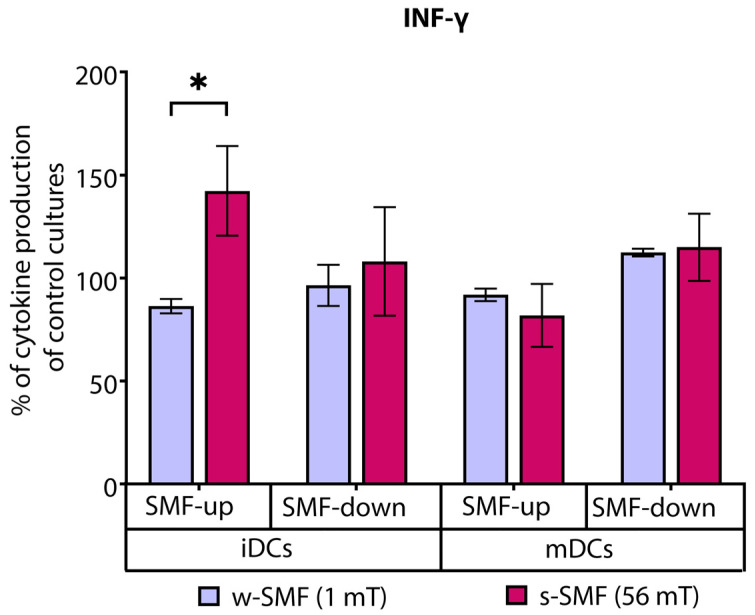
Release of IFN-γ by CD4+ T cells, stimulated by DCs in the cultures that were continuously exposed to 1 mT or 56 mT SMF. Results are expressed as a percentage of cytokine production of control cultures, and bars are standard error of the mean for *n* = 3 independent experiments; * *p* < 0.05. iDC: immature dendritic cells, mDC: mature dendritic cells, SMF: static magnetic field, w-SMF: weak static magnetic field, s-SMF: strong static magnetic field, up: upward, and down: downward.

The effects of an SMF on DC-induced T-cell cytokine production varied between tested cytokines, depending on the SMF strength and activation stage of DCs (immature/mature DC). T cells stimulated by iDCs in the presence of the upward-oriented 56mT SMF produced higher levels of IFN-γ, which were 42.35% increased in comparison to unexposed control cells. On the other hand, the exposure of these cells to the 1 mT SMF of the same orientation was followed by a lower production of IFN-γ, which was 13.62% decreased compared to control cells. Comparison of fold change values, expressed as a ratio of cytokine levels between exposed and unexposed cells, revealed that elevation of IFN-γ was significantly higher when the cells were exposed to the upward-oriented 56 mT versus 1 mT SMF (*p* < 0.5). However, significant effects on the production of IFN-γ were not demonstrated when cells were exposed to downward-oriented SMFs of different strengths, nor when T cells were stimulated by mature DCs (Figure 4).

**Figure 5:**
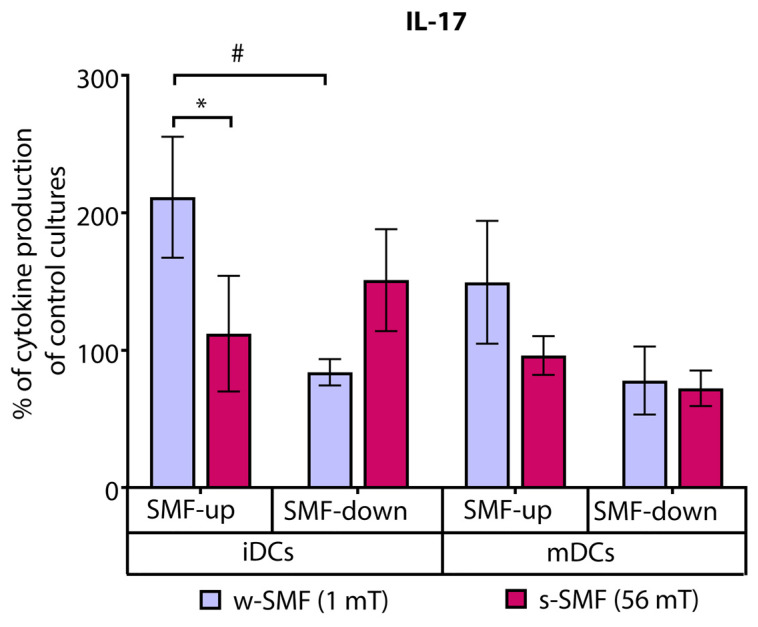
Release of IL-17 by CD4+ T cells stimulated by DCs in the cultures that were continuously exposed to 1 mT or 56 mT SMF. Results are expressed as a percentage of cytokine production of control cultures, and bars are standard error of the mean for *n* = 3 independent experiments; * *p* < 0.05, iDC (1 mT SMF-up) compared to iDC (56 mT SMF-up); ^#^ *p* < 0.05, iDC (1 mT SMF-down) compared to iDC (1 mT SMF-up). iDC: immature dendritic cells, mDC: mature dendritic cells, SMF: static magnetic field, w-SMF: weak static magnetic field, s-SMF: strong static magnetic field, up: upward, and down: downward.

In contrast, the levels of IL-17 were increased by 111.37% compared to the control cell culture when T cells and iDCs were exposed to the upward-oriented weaker SMF (1 mT). The elevation of IL-17 production was significantly higher when the cells were exposed to the 1 mT versus 56 mT SMF (*p* < 0.05). Interestingly, an opposite but not significant effect was observed when T cells and iDCs were exposed to the downward-oriented SMF. Comparison of IL-17 fold change values of the cells exposed to the upward-oriented and downward-oriented 1 mT SMFs revealed a significant opposite effect of differently oriented SMFs (*p* < 0.05) (Figure 5).

IL-4 production was not significantly modified in any of the tested conditions.

**Figure 6:**
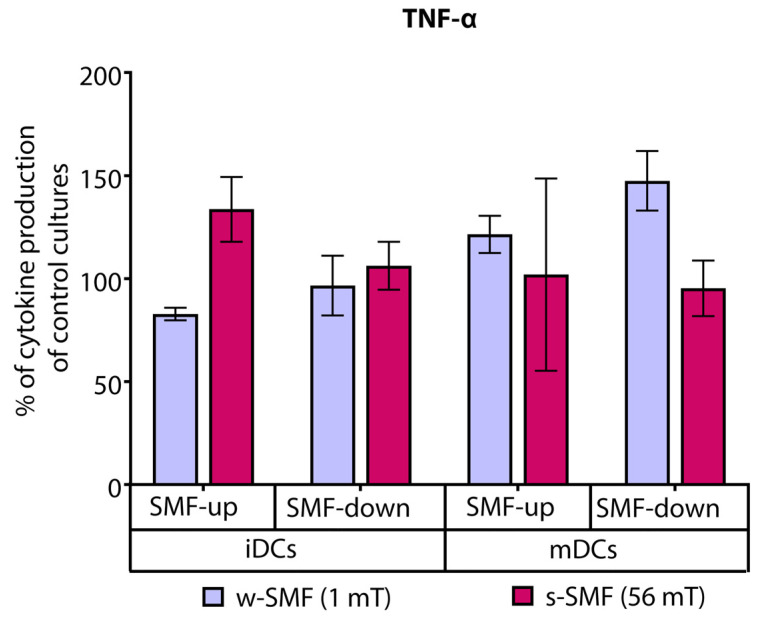
Release of TNF-α by CD4+ T cells stimulated by DCs in the cultures that were continuously exposed to 1 mT or 56 mT SMF. Results are expressed as a percentage of cytokine production of control cultures, and bars are standard error of the mean for *n* = 3 independent experiments. iDC: immature dendritic cells, mDC: mature dendritic cells, SMF: static magnetic field, w-SMF: weak static magnetic field, s-SMF: strong static magnetic field, up: upward, and down: downward.

Analysis of proinflammatory cytokines revealed that T cells stimulated with iDCs and exposed to the upward-oriented 56 mT SMT produced elevated levels of TNF-α (21.53% increased values) than the cells exposed to the 1 mT SMT of the same orientation (17.15% decreased values). Interestingly, the opposite trend was observed when the T cells were stimulated with mDC and exposed to the downward-oriented SMF. The levels of TNF-α were 47.50% increased in comparison to unexposed control cells when the cells were exposed to the 1 mT SMF. At the same time, they were unchanged or slightly decreased with 56 mT exposure. The observed difference in TNF-α fold change values recorded for 1 mT and 56 mT approached the criterion for significance (*p* = 0.075) (Figure 6).

**Figure 7:**
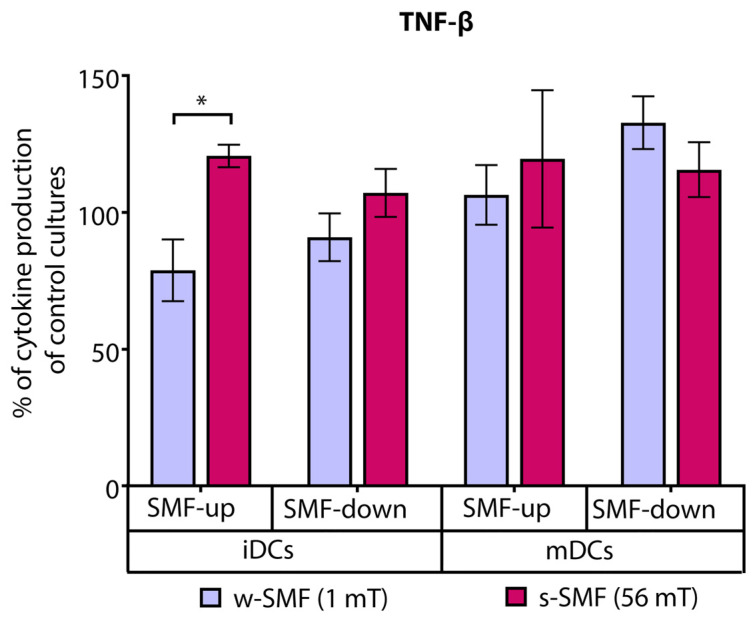
Release of TNF-β by CD4+ T cells stimulated by DCs in the cultures that were continuously exposed to 1 mT or 56 mT SMF. Results are expressed as a percentage of cytokine production of control cultures, and bars are standard error of the mean for *n* = 3 independent experiments; * *p* < 0.05, iDC (1 mT SMF-up) compared to iDC (56 mT SMF-up). iDC: immature dendritic cells, mDC: mature dendritic cells, SMF: static magnetic field, w-SMF: weak static magnetic field, s-SMF: strong static magnetic field, up: upward, and down: downward.

TNF-β production was elevated in the cultures of T cells iDCs exposed to the upward-oriented 56 mT SMF (20.69% increase) and decreased when the cells were exposed to the upward-oriented 1 mT SMF (21.13% decrease). The observed differences in relative values reflecting TNF-β secretion were significant (*p* < 0.05) (Figure 7).

For other tested cytokines, including IL-1β, IL-6, IL-8, and anti-inflammatory cytokine IL-10, no statistical significance was found compared to unexposed cells.

### 3.2. Cell Proliferation and Viability

To further evaluate whether the observed differences in cytokine levels could be related to different activation rates and the viability of stimulated T cells, we quantified the proliferation and analyzed cell death of T cells.

**Figure 8:**
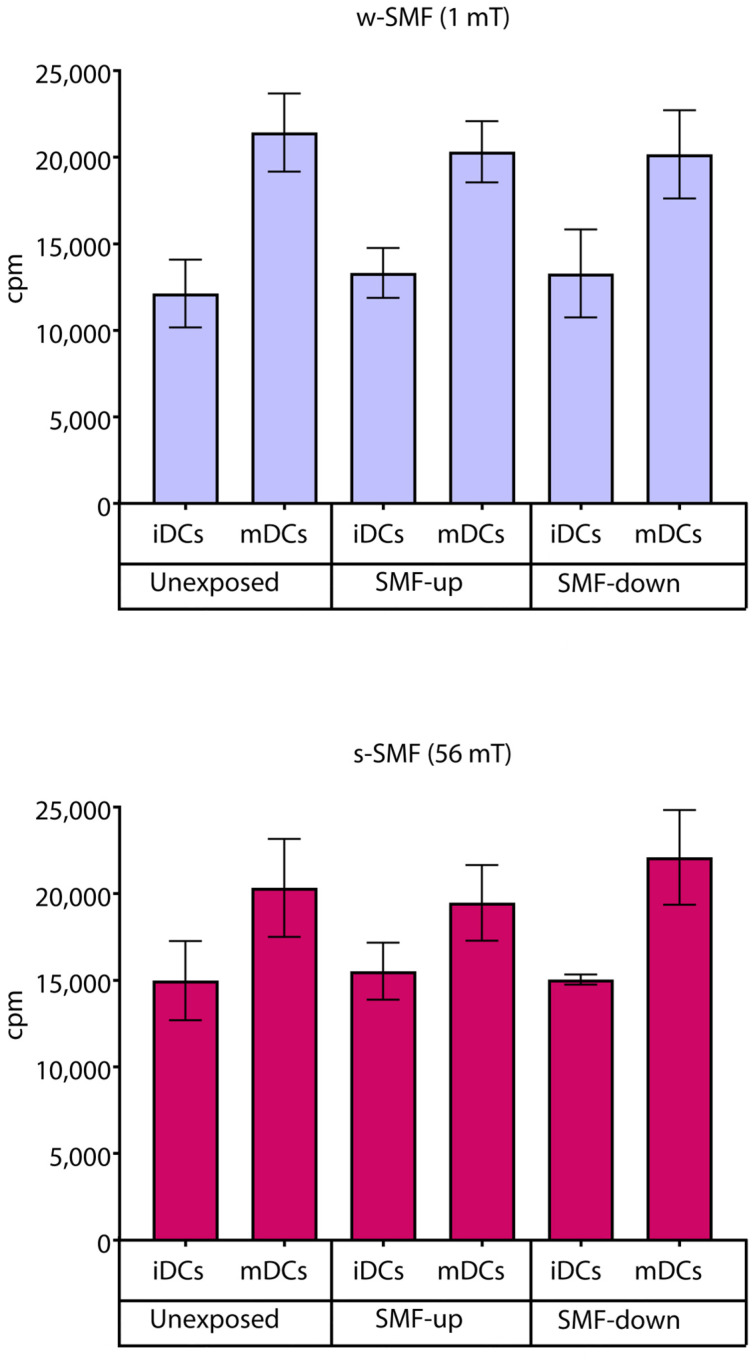
Proliferation of T cells stimulated by DCs in the cultures that were continuously exposed to 1 mT or 56 mT SMF. Results are expressed as counts per minute (cpm), and bars are standard deviations of the mean for *n* = 3 independent experiments. iDC: immature dendritic cells, mDC: mature dendritic cells, SMF: static magnetic field, w-SMF: weak static magnetic field, s-SMF: strong static magnetic field, up: upward, and down: downward.

Cell proliferation was analyzed in an allogeneic mixed leukocyte reaction separately for the cells exposed to the 1 mT and 56 mT SMFs. As expected, the results obtained demonstrated a higher proliferative response when T cells were stimulated with mDCs. However, there were no significant changes in cell proliferation in the stimulated cells regardless of the strength and orientation of the SMF they were exposed to, suggesting the activation rate of T cells was unaffected by the tested SMF (Figure 8).

**Figure 9:**
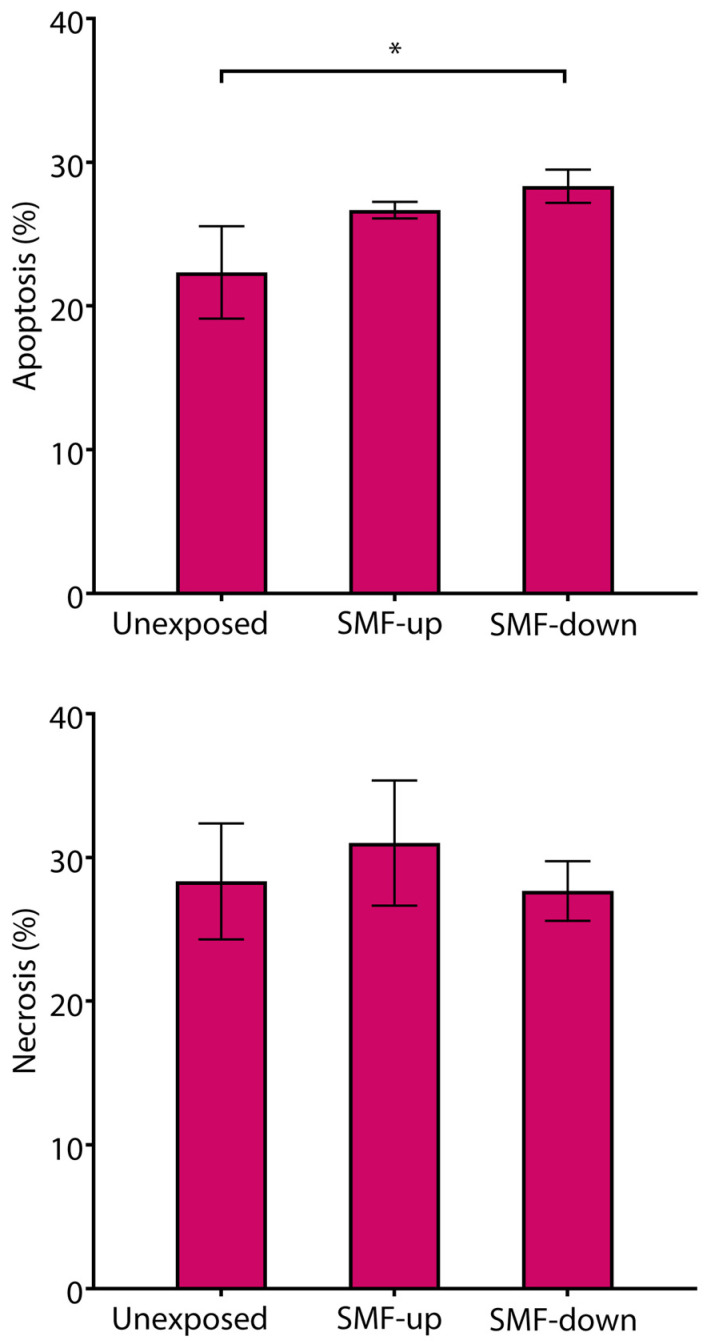
Viability of T cells continuously exposed to 56 mT SMF. Results are expressed as a percentage of apoptotic cells and necrotic cells, and bars are standard deviations of the mean for *n* = 3 independent experiments. * *p* < 0.05, compared to control cells. SMF: static magnetic field, up: upward, and down: downward.

Cell death was analyzed using flow cytometry, quantifying the number of cells with hypodiploid nuclei and cells with damaged cell membranes, which represent apoptotic and necrotic cells, respectively. After 6 days of T cell exposure to 56 mT SMFs of different orientations, the number of apoptotic and necrotic cells was around or below 30% of the total cells, demonstrating no cytotoxic effect of the SMF. A slight but significant difference was observed only between control cells and cells exposed to the downward-oriented SMF, demonstrating a higher number of apoptotic cells in the SMF-exposed cultures (control vs. downward-orientated SMF: 22.33 ± 3.21 vs. 28.33 ± 1.15; *p* < 0.05). However, analysis of necrotic cells did not show any difference in T cell viability between tested conditions (Figure 9).

## 4. Discussion

Although the effect of an SMF of moderate intensity has been previously shown to have a certain influence on biological systems, there are limited investigations on the impact of a moderate SMF at the cellular level, including immune cells [1,2,15].

A few studies have demonstrated the modulatory effect of a magnetic field on the production of cytokines by different cell types. It has been shown that the ability of PBMCs to produce certain cytokines may be altered by acute exposure to a strong 0.5 T SMF by an MRI machine [4,6]. The acute exposure was also tested on pure human CD4+ T cells, but these cells were activated with mitogens such as phytohemagglutinin [7]. We focused on CD4+ T cells, but not total CD3+ T cells considering the pivotal role of CD4+ T cells in orchestrating immune responses by differentiating into T helper cell subsets, secreting distinct sets of cytokines [16]. Furthermore, CD4+ T cells play critical roles in the pathogenesis of immune-mediated diseases and showing magnetic field modulation of their function could open a new therapeutical approach in the treatment of pathological conditions. Moreover, recently it has been shown that CD4+, but not CD8+, chimeric antigen receptor (CAR) T cells are the main drivers of cytokine release syndrome, which is known as the side effect of CAR T cell immunotherapy which causes the most significant treatment-related toxicity [17]. Furthermore, CD4+ and CD8+ T cells could respond differently to allogeneic stimulation, such as a delayed activation of CD8+ T cells in a mixed leucocyte reaction [18], suggesting that assessment of the magnetic field effect on purified T cell subsets has an advantage over assessment of the total T cell population.

The effects of a moderate SMF were further tested on fibroblasts that were stimulated with lipopolysaccharide and exposed for 12 h to an SMF [19]. However, according to our knowledge, there are no published data about the effects of a continuous and moderate SMF on lymphocytes activated by DCs, and there are no studies in which lymphocytes were exposed to SMFs of different polarities. Therefore, in this study, we investigated the changes in in vitro cytokine production in cultures consisting of purified human T lymphocytes activated by monocyte-derived DCs. The changes were analyzed after continuous exposure to vertical homogeneous SMFs of moderate intensity (1 mT and 56 mT) and of different orientations.

Assuming that SMF exposure has the potential to alter interleukine production by activated T cells, we first tested the effect on the secretion of three interleukins that belong to different activation pathways of T cells: IFN-γ, which is inherent to the T helper 1 (Th1) cells predominantly involved in cellular immune response; IL-4, which is a hallmark of T helper 2 (Th2) cells, which are involved in the humoral immune response; and IL-17, which is characteristic of T helper 17 (Th17) cells, which play a crucial role in maintaining protection of mucosal barriers and are implicated in chronic and autoimmune inflammatory disorders [20,21]. We further examined pro- and anti-inflammatory interleukins.

We showed that the upward-oriented 56 mT SMF significantly increases IFN-γ production in the cultures where the cells were stimulated by immature DCs. In contrast, IL-17 production was increased when cells were exposed to the weaker SMF (1 mT). The effect was also observed, like with IFN-γ production, in cultures with immature DCs. IL-4 concentration showed no statistically significant modification in any of the tests.

It was very intriguing to note that T cells activated by mature DCs produced more TNF-α in response to the downward-oriented weaker (1 mT) SMF. When the 1 mT SMF is pointed upward, TNF-β production in T cells activated by immature DCs is reduced. IL-1, IL-6, and IL-8 production were unaffected. Additionally, the production of the anti-inflammatory cytokine IL-10 was unaffected.

Previous research has demonstrated that 2 h of exposure to a 0.5 T SMF increased the production of IFNγ and IL-4 in PBMCs. The impact was diminished with PHA stimulation [6]. However, it has been shown that 0.5 mT exposure for 2 h on subsets of human CD4+ T cells (naive and memory cells) decreased the release of IFN-γ, and this effect was rapidly reversible when the analyses were repeated after prolonged in vitro culture [7].

Short-term high SMF exposure did not change the generation of proinflammatory cytokines (1–4 h) [4,6]. The release of the proinflammatory cytokines IL-6, IL-8, and TNF-a from macrophages and IL-6 from lymphocytes, however, is significantly inhibited by exposure to a strong, inhomogeneous SMF continuously for up to 24 h. The amount of the anti-inflammatory cytokine IL-10 produced by lymphocytes and macrophages was increased [5].

SMFs have been found to affect tumor cells, particularly through their inhibitory actions. Cancer treatment involves a combination of several therapies due to the toxicity and resistance of individual anticancer medications. One of the techniques is the interaction of magnetic fields with anticancer medications. This combination provides a new strategy for the effective treatment of cancer. Additionally, since we can use a permanent magnet instead of leads and an external energy source, SMF stimulation is better suited for long-term local healing. An SMF’s beneficial impacts could have further applications [22,23,24].

There is growing evidence that SMFs can affect cell proliferation, although different findings have been published. Certain studies indicate that SMFs do not have an impact on cell proliferation or cell cycle [25,26]. The others, however, indicate that SMFs, either by themselves or in combination with chemotherapy medications or radiation, demonstrate potential benefits for the inhibition of cancer growth. Furthermore, has been proven previously that an ultra-high SMF can cause immunosupression through disrupting B-cell peripheral differentiation and consequently the occurrence of many immunologic disorders [22,23,27,28]. As a result, it is still unclear exactly how long-term exposure to SMFs affects human bodies. It was reported that a 4.75 T SMF significantly reduced the proliferation of leukemia cells, but without an effect on the proliferation of normal lymphocytes [4]. Additionally, it has been shown that a 7 T SMF has the ability to inhibit the proliferation of specific cancer cell lines [13]. On the other side, there are studies that have revealed that even 10 T-strong SMFs do not cause evident alteration in non-cancer cells such as Chinese hamster ovary cell lines or human fibroblast cells [29,30,31]. Furthermore, recent studies have determined that SMFs, by promoting its paracrine effect, accelerate bone tissue regeneration and improves its quality [32], repair various ischemic tissue injuries by promoting angiogenesis and tissue regeneration [33], and enhance the repair of arthritic cartilage by promoting chondrogenesis and the migration of stem cells [34]. These approaches have wide-ranging uses in the regeneration of bone and skin tissue, the treatment of limb ischemia and myocardial injury, and therapy for diabetic wounds [33]. These findings demonstrate that the varied cellular responses to SMFs are influenced to a large extent by cell type. The effects of 1 T on 15 different cell lines, including 12 human cell lines (5 non-cancer and 7 solid cancer) and 3 rodent cell lines, revealed that 1 T SMF has no discernible impact on the cell cycle or cell death. However, the reduction in cell numbers in six out of seven solid human cancer cell lines was seen at a higher cell density. Both cell type and cell density had an apparent influence on SMF effects. The results showed that a 1 T SMF can inhibit the cell growth of most human solid cancer cell lines but not non-cancer cell lines [27]. Seven days of prolonged exposure to a 0.5 Tesla SMF reduced proliferation and cytokine production (TGF-β1, IGF-1, VEGF) in adipose-derived stem cells [35]. Exposure to an SMF up to 24 mT decreases the mesenchymal stem cell proliferation rate [36]. It was shown that different magnetic field directions produce divergent effects on cancer cell numbers of 12 different cell lines (5 human solid tumor cell lines, 4 human non-cancer cell lines, and 2 human leukemia cell lines, as well as the Chinese hamster ovary cell line), when the cells were exposed to 0.2–1 T SMFs with different magnetic field directions [37]. It was demonstrated that an SMF at 15 mT downregulated the expression of osteoclast-specific transcription factors in mouse bone marrow-derived macrophage cells, and with that impact, an SMF could contribute to the inhibition of bone resorption and osteoclast formation. They may be employed in the treatment of osteolytic conditions like osteoporosis and rheumatoid arthritis [38].

As a result, it is challenging to directly compare the publications cited here because of the differences in the static magnetic field intensity, exposure period, and subject exposure. Not only do the experimental results in the literature vary, but they even run counter to one another. It is intriguing because reports have shown variances between magnetic field directions that are in opposition [3,10,11]. By interfering with the inflammatory and anti-inflammatory cytokines, SMFs were shown to have both stimulatory and inhibitory effects on immune system response. Evidence also suggests that long-term exposure to SMFs may reduce adaptive immunological responses, particularly in the Th1 fraction of cells [38]. In order to better understand the biological impacts of SMFs, we have now demonstrated that it is also important to consider the various polarities of SMFs with reference to cytokine production. However, our study had a few limitations. Firstly, we acknowledge that our study is limited mainly to ELISA data and lacks mechanistic insight, which can limit the ability to validate the results effectively. However, this is the first time that it has been proven that different SMF directions induce distinct effects on cytokine production by lymphocytes activated with human DCs.

## 5. Conclusions

In conclusion, our results show that different magnetic field directions produce different effects on cytokine production by lymphocytes activated with human DCs. This could help achieve a better understanding of the biological effects of SMFs and, besides intensity, add a new component in differentiating a possible beneficial effect of an SMF on the human body.

## Data Availability

Authors give consent for all data and materials to be available.
